# Dietary fiber enhances milk yield in plateau dairy cows via activation of the rumen microbiota-mammary gland axis

**DOI:** 10.3389/fvets.2025.1654799

**Published:** 2025-08-12

**Authors:** Bin Li, Dongxu Wen, Ziwei Zhou, Quji Suolang, Wangmu Siwang, Lamu Kangji, Tuoxian Tang, Zhenjiang Liu, Yachun Wang

**Affiliations:** ^1^School of Animal Science and Technology, Ningxia University, Yinchuan, China; ^2^Xizang Autonomous Region Academy of Agricultural and Animal Husbandry Sciences, Key Laboratory of Animal Genetics and Breeding on Tibetan Plateau, Ministry of Agriculture and Rural Affairs, Institute of Animal Husbandry and Veterinary Science, Lhasa, China; ^3^School of Life Sciences, Jilin University, Changchun, China; ^4^Xizang Autonomous Region Institute of Science and Technology Information, Lhasa, China; ^5^Department of Biology, University of Pennsylvania, Philadelphia, PA, United States; ^6^Laboratory of Animal Genetics, Breeding and Reproduction, Ministry of Agriculture of China, National Engineering Laboratory of Animal Breeding, College of Animal Science and Technology, China Agricultural University, Beijing, China

**Keywords:** fiber, milk yield, LXR signaling, hypoxia, mammary gland

## Abstract

Milk yield in high-altitude regions such as the Qinghai-Tibet Plateau is low due to hypoxic stress and impaired mammary gland function. This study aims to determine whether a fiber-supplemented diet could increase milk yield in plateau dairy cows through modulating rumen microbiota and downstream metabolic signaling. Holstein cows were assigned to diets containing either *Brassica rapa L.* or an aquatic plant with a high neutral and acid-detergent fiber content. Milk yield and rumen metabolites were analyzed, and additional functional assays were performed using bovine mammary epithelial cells (BMECs) cultured under hypoxic conditions. The *Brassica rapa L.* supplementation significantly increased milk yield, which was associated with elevated levels of fiber-derived metabolites, including cholesterol valerate and 5-oxoeicosapentaenoic acid. These metabolites activated liver X receptor signaling in mammary cells under hypoxia, as validated by proteomic analysis and LXRα expression. Gene enrichment analysis revealed that LXR signaling was associated with lipid metabolism and cellular adaptation to low oxygen. These results support a fiber-microbiota-mammary axis, showing that fiber supplementation enhances milk yield through metabolic signaling. Moreover, this study presents a sustainable and feasible method to enhance milk production in ruminants under environmental stress.

## 1 Introduction

Dairy cows rely heavily on the function of the rumen, a specialized fermentation chamber that enables the digestion of fibrous plant materials into absorbable energy-rich metabolites. This complex organ hosts a diverse community of microorganisms, including bacteria, archaea, fungi, and protozoa that collaborate to degrade cellulose and hemicellulose, making the rumen a critical determinant of ruminant nutritional efficiency and overall productivity. Improving milk yield in dairy cows remains a core objective in animal science, particularly under challenging environments such as high-altitude regions, where metabolic stress and hypoxia limit productivity. Recent research highlights that nutritional modulation of host metabolism extends beyond nutrient absorption and includes signal transduction through microbially derived metabolites. The stability and metabolic output of the rumen microbiota are closely linked to host performance traits such as growth rate and milk production (1, 2). Milk yield in high-altitude regions, such as the Qinghai-Tibet Plateau, is significantly lower than in lowland areas. This is largely attributed to chronic hypoxic stress, which affects systemic oxygen delivery and cellular energy metabolism, particularly in oxygen-sensitive tissues such as the mammary gland (3). Mammary epithelial cells under hypoxia show reduced proliferation and impaired lipogenesis, limiting the cow’s capacity for sustained milk secretion (4). Despite their adaptation to altitude, indigenous dairy cows often show suboptimal lactation, partly due to limited forage quality and altered energy metabolism. Strategies to improve fiber utilization and enhance systemic metabolic signaling are urgently needed but remain understudied in these regions.

Metabolite-sensing nuclear receptors such as LXRα, PPARs, and FXR have emerged as key regulators of tissue-specific metabolic programs in response to dietary and environmental inputs. Among these, LXRα plays a central role in lipid handling, oxidative stress, and energy homeostasis, and may integrate dietary signals to support lactation under hypoxia. While nutritional strategies such as increasing energy or protein supply have been explored, less attention has been given to the role of dietary fiber as a functional signal modulator. Traditionally considered a bulking agent, fiber is now recognized as a key substrate for microbial fermentation, producing short-chain fatty acids and lipid-derived metabolites with regulatory effects on host metabolism (5, 6). Certain microbial metabolites, such as oxylipins and sterol derivatives, can activate nuclear receptors like the liver X receptor (LXR), which controls lipid synthesis, cellular homeostasis, and mammary gland differentiation (7, 8). Emerging evidence suggests the existence of a rumen microbiota-mammary gland signaling axis, through which dietary inputs influence microbial metabolite profiles that regulate host tissue metabolism. However, this concept remains largely untested in lactation under hypoxic stress, especially in a high-altitude environment where the mammary gland may be more sensitive to metabolic changes. Moreover, the effect of fiber-derived microbial metabolites on lactation remains poorly understood, particularly through LXR signaling pathways.

In addition to its role in ruminal fermentation, dietary fiber has been shown to modulate immune responses and epithelial barrier function through microbial-derived metabolites such as short-chain fatty acids (SCFAs) and secondary bile acids. These systemic effects may contribute to mammary gland integrity and function during lactation. Recent studies have demonstrated that high-fiber diets alter rumen microbial communities and increase the production of volatile fatty acids and bioactive lipids that potentially influence peripheral tissues (9, 10). Nonetheless, direct evidence linking specific microbial metabolites to molecular changes in the mammary gland remains limited, and few studies have integrated rumen metabolomics with functional proteomic analyses of mammary epithelial cells. Therefore, we hypothesized that supplementing plateau dairy cows with fiber-rich diets would modulate the rumen microbiota and enhance the production of lipid-derived metabolites that activate LXR signaling in the mammary gland under hypoxic conditions, thereby improving lactation performance. Metabolomics of rumen microbiota and proteomics of mammary epithelial cells under hypoxia were utilized to test our hypothesis. This study provides a sustainable and feasible strategy for improving dairy productivity in high-altitude environments.

## 2 Materials and methods

### 2.1 Animals, diet, and experimental design

The experiment was conducted in the Lalu wetland, situated in the northwest of Lhasa City, Tibet Autonomous Region (29°39′-29°42′N, 91°03′-91°06′E, altitude 3,645 m) (11). Thirty periparturient Holstein cows were assigned to three treatment groups (*N* = 10 / group) according to a randomized complete block design of parity (first pregnancy), milk yield (26.1 ± 1.32 kg/d), and body weight (550.3 ± 13.9 kg). (1) The Control group (Con): Normal feeding according to perinatal standards. The normal diets were composed of 75% concentrate and 25% roughage as described in our previous paper (12). (2) *Brassica rapa L*. feeding (Bra): Cows were fed an additional 20 g/day of lyophilized *Brassica rapa L.* powder (The main component is polysaccharides, with a content of 32.84%) to standard feeding. (3) Aquatic plants (Aq): fed with the same amount of aquatic plant silage inoculated with Yeast (YS). Aquatic plants (mainly *Sparganium stoloniferum*, reed, cattail, etc.) were harvested and collected in July 2024. The animal experiment was conducted following the guidelines of the Animal Care and Use Committee of the Institute of the College of Life Sciences, Jilin University [Approval No. (2024) Shen YNPZSY No. (1102)].

### 2.2 Diet and chemical analysis

The silage and diet material underwent a 72-h drying process in the oven at 65°C. Subsequently, the dried samples were ground and sieved through a 1 mm sieve for further analysis. Crude fiber components, including acid detergent fiber (ADF) and neutral detergent fiber (NDF), were measured following the methodology outlined by Yang et al. (12).

### 2.3 Identification of rumen fluid metabolites

Metabolites in the rumen fluid were identified using liquid chromatography–tandem mass spectrometry (LC–MS/MS). The sample was removed from the −80°C freezer and thawed on ice until fully defrosted (all subsequent steps were performed on ice). After vortexing for 10 s to mix thoroughly, 200 μL of the sample was transferred to a centrifuge tube. An equal volume of 20% acetonitrile-methanol internal standard extract was added, followed by vortexing. The mixture was centrifuged at 12,000 × g for 10 min at 4°C, and the supernatant was collected and concentrated to dryness. The resulting powder was reconstituted in 100 μL of 70% methanol solution, sonicated in an ice-water bath for 10 min, and centrifuged at 12,000 × g for 3 min at 4°C. Finally, 80 μL of the supernatant was collected for LC–MS/MS detection (SCIEX, USA). A detailed description of the chromatographic and mass spectrometric conditions was shown in a recent report (13).

### 2.4 Western blotting

Total 30 μg of each protein sample was loaded onto a polyacrylamide gel for electrophoretic separation and transferred onto a polyvinylidene fluoride membrane. The membrane was incubated with the following primary antibodies: anti-LXR-alpha antibody (1:1,000, Abcam, Cat#ab3585), and anti-*β*-actin antibody (1:1,000, Cell Signaling Technology, Cat# 4970) was used as a loading control. After blocking with 5% non-fat milk in PBST (PBS containing 0.1% Tween-20), the membrane was incubated with the primary antibody overnight at 4°C, followed by three washes with PBST. The membrane was then incubated with HRP-conjugated secondary antibodies, including HRP-conjugated anti-rabbit IgG antibody (1:4,000, Cell Signaling Technology, Cat# 7074). Protein bands were detected using a chemiluminescence imaging system (Tanon, Shanghai, China), and grayscale analysis of the blot images was performed using ImageJ software.

### 2.5 Cell culture

The bovine mammary epithelial cell line BME-UV1 was cultured in Dulbecco’s Modified Eagle Medium/Nutrient Mixture F-12 (DMEM/F12; Gibco, USA) supplemented with 10% fetal bovine serum (FBS; Gibco), 5 μg/mL insulin, 5 ng/mL epidermal growth factor (EGF), 1 μg/mL insulin-like growth factor I (IGF-I), 5 μg/mL transferrin, 100 U/mL penicillin, and 100 μg/mL streptomycin. Cells were maintained at 37°C in a humidified atmosphere containing 5% CO₂. When reaching approximately 80–90% confluence, cells were detached using 0.25% trypsin–EDTA and passaged for experimental use. For the Hypoxia group, cells were transferred to a hypoxia camber which was filled up with 1% O₂, 5% CO₂, 94%N₂, and the other plate of cells was placed on side of the chamber as the Normoxia group.

### 2.6 Protein expression, localization, and correlation analysis

Bovine mammary epithelial cells (BME-UV1) were cultured under normoxic (21% O₂) or hypoxic (1% O₂) conditions, or treated with 200 μM CoCl₂ to mimic hypoxia. For protein expression analysis, cells were harvested at 0 h, 4 h, and 8 h after treatment, and total protein was extracted for Western blot using anti-LXRα antibodies (Abcam, ab41902). *β*-actin was used as the loading control. Densitometric analysis was performed using ImageJ. For subcellular localization, cells were transfected with GFP-tagged bovine LXRα using Lipofectamine 3,000 (Thermo Fisher), incubated under hypoxia or normoxia for 8 h, and visualized under a confocal microscope (Leica TCS SP8). Nuclear localization was determined by overlap with DAPI staining.

Correlation analysis between hypoxia scores and NR1H3 (LXRα) mRNA expression was performed using transcriptomic data from the TCGA breast cancer cohort (BRCA, *n* = 1,100). Hypoxia scores were calculated based on a published 15-gene hypoxia signature, and correlation was assessed using Pearson’s coefficient (R) in R (version 4.2.2).

### 2.7 HEK293 cell culture and transcriptomic profiling of LXRα overexpression

HEK293 cells (human embryonic kidney 293, ATCC CRL-1573) were cultured in Dulbecco’s Modified Eagle Medium (DMEM, Gibco) supplemented with 10% fetal bovine serum (FBS, Gibco) and 1% penicillin–streptomycin at 37°C in a humidified incubator with 5% CO₂. Cells were seeded in 6-well plates and transfected with a pcDNA3.1 expression vector encoding bovine LXRα or an empty vector (EV) control using Lipofectamine 3000 (Invitrogen) according to the manufacturer’s instructions. At 48 h post-transfection, total RNA was extracted using TRIzol reagent (Invitrogen) and purified using the RNeasy Mini Kit (Qiagen). RNA-seq libraries were prepared using the NEBNext Ultra RNA Library Prep Kit (NEB), and sequencing was performed on an Illumina NovaSeq 6000 platform with 150 bp paired-end reads. Raw data were quality-checked using FastQC, aligned to the human genome (hg38), and analyzed using DESeq2 for differential gene expression. Genes with adjusted *p*-values < 0.05 and |log₂FC| > 1.5 were considered significantly regulated. KEGG pathway enrichment was performed using the DAVID tool to identify biological processes associated with LXRα activation.

### 2.8 Bioinformatics analysis

Raw sequencing data (Fastq format) were subjected to quality control using fastp, removing adapter sequences, low-quality reads, and poly-N-containing reads, while calculating Q20, Q30, and GC content. High-quality clean reads were aligned to the mouse reference genome (GRCm39) using HISAT2 (v2.0.5). Gene expression levels were quantified with FeatureCounts (v1.5.0-p3) and converted into FPKM values. Differential expression analysis was performed using the DESeq2 package, applying cutoff criteria of |log₂FoldChange| > 1 and *p* < 0.05. Gene Ontology (GO) functional enrichment and KEGG (Kyoto Encyclopedia of Genes and Genomes) pathway enrichment analyses were performed using the ClusterProfiler R package, with a significance threshold set at p.adjust < 0.05.

### 2.9 Co-immunoprecipitation assay

HEK293 cells were seeded in 6-well plates and transiently transfected with expression constructs encoding FLAG-tagged bovine LXRα and HA-tagged bovine HIF-1α using Lipofectamine 3,000 (Invitrogen). After 24 h, cells were treated with 100 μM CoCl₂ for 8 h to simulate hypoxia. Total protein lysates were extracted using RIPA buffer containing protease inhibitors. For immunoprecipitation, 500 μg of lysate was incubated with anti-FLAG or anti-HA antibody (Cell Signaling Technology) overnight at 4°C, followed by incubation with protein A/G magnetic beads for 2 h. Beads were washed and eluted samples were analyzed by SDS-PAGE and Western blot using reciprocal antibodies. Input and IP samples were probed with anti-FLAG and anti-HA antibodies to detect LXRα and HIF-1α, respectively.

### 2.10 Statistical analysis

All experimental data are presented as the mean ± standard error of the mean (SEM) from at least three independent experiments. Statistical analyses were performed using GraphPad Prism version 10.3 (GraphPad Software, San Diego, CA, USA). Unpaired Student’s *t*-tests were conducted to compare the two groups. The statistical significance level of *p* < 0.05 was considered significant. Before performing *t*-tests, data were checked for normality and homogeneity of variances. All results were graphically presented using GraphPad Prism software, with statistically significant differences indicated by asterisks or other appropriate symbols (*p* < 0.05, *p < 0.01, **p < 0.001).

## 3 Results

### 3.1 High-fiber diets significantly increased milk yield

**Figure 1:**
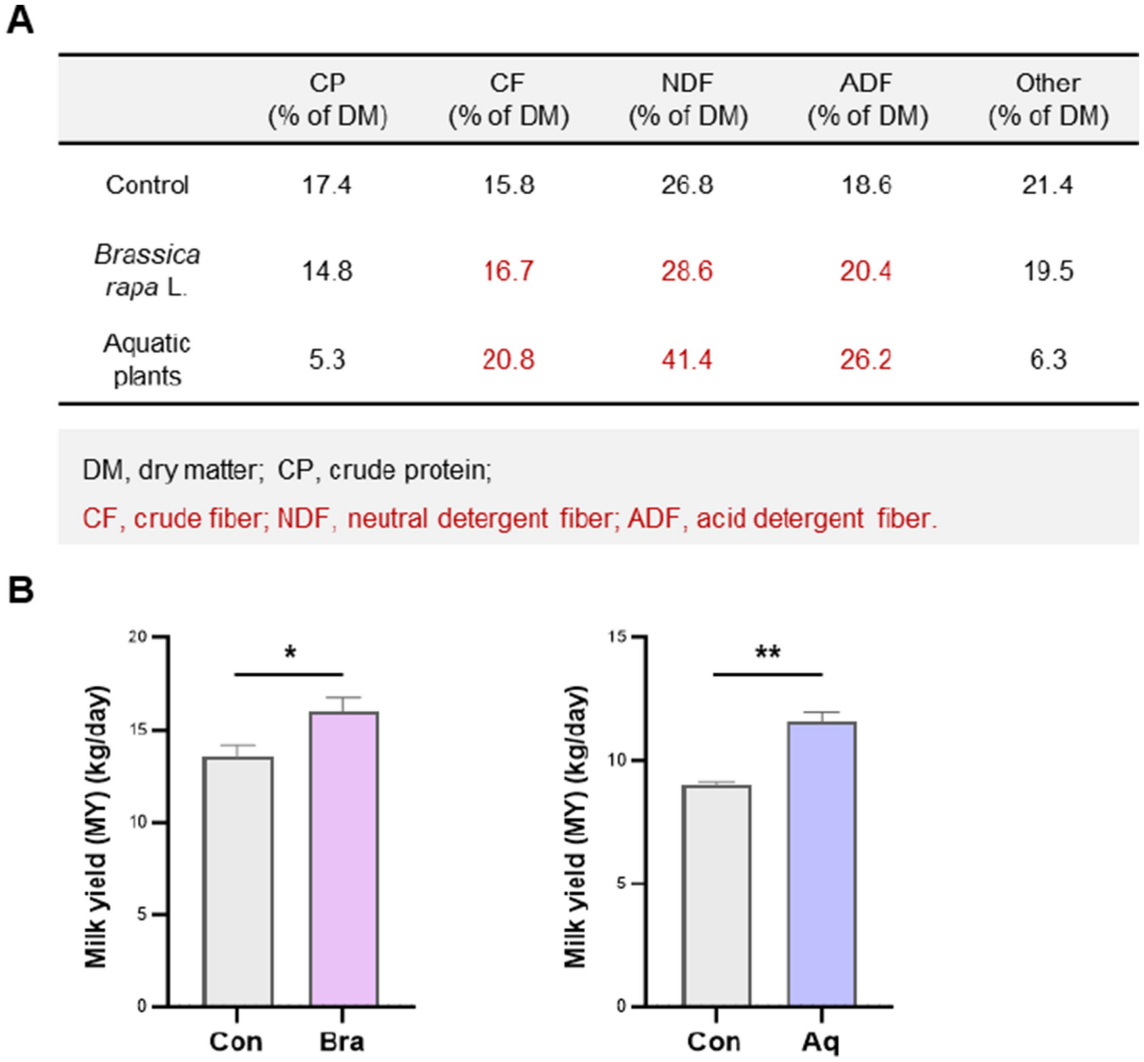
Dietary fiber from *Brassica rapa L.* improves milk yield in plateau dairy cows. **(A)** Nutritional composition of the control, *Brassica rapa L.*, and aquatic plant dietss. **(B)** Daily milk yield in cows fed each diet. *Brassica rapa L.* and aquatic plant diets significantly increased milk production compared to the control group (*p* < 0.05), with the *Brassica rapa L*. group showing the highest yield. Data are presented as mean ± SEM. * *p* < 0.05; ** *p* < 0.01.

As shown in Figure 1, the *Brassica rapa L.* and aquatic plant diets provided substantially higher levels of structural fiber than the control diet. Specifically, the *Brassica rapa L.* diet contained 16.7% crude fiber (CF), 28.6% neutral detergent fiber (NDF), and 20.4% acid detergent fiber (ADF), while the aquatic plant diet contained 20.8% CF, 41.4% NDF, and 26.2% ADF. In contrast, the control diet had lower levels of these components, at 15.1, 27.0, and 19.1%, respectively. These differences resulted in a total fiber-related content (CF + NDF + ADF) of approximately 65.7% of dry matter in the *Brassica rapa L*. diet and 88.4% in the aquatic plant diet. In addition to higher fiber content, both fiber-rich diets significantly increased milk yield compared to the control group (*p* < 0.05). Despite the higher fiber content in the aquatic plant diet, milk yield was greater in the *Brassica rapa L.* group, suggesting that fiber composition may have a more critical impact than total fiber content.

### 3.2 Fiber feeding alters rumen metabolite profiles, enhancing lipid-associated metabolites

**Figure 2:**
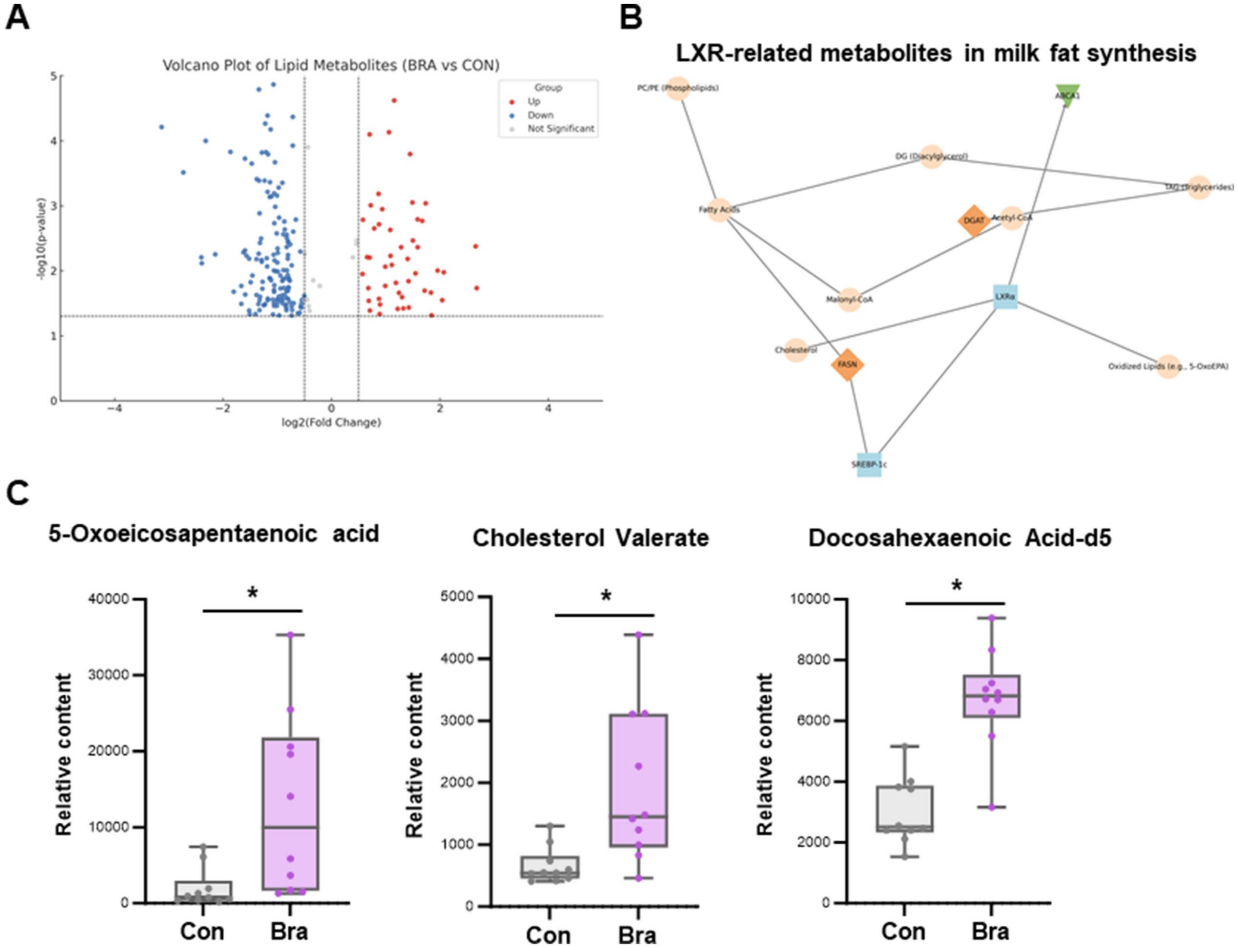
Rumen metabolomic profiling reveals increased lipid-associated signaling metabolites under fiber feeding. **(A)** Volcano plot showing differentially abundant lipid metabolites between *Brassica rapa* and control groups. Red and blue dots represent significantly upregulated and downregulated metabolites, respectively. **(B)** Metabolic network highlighting the association between lipid metabolites and liver X receptor alpha (LXRα), with connections to oxidized lipids, cholesterol, and downstream effectors such as SREBP-1c. **(C)** Boxplots showing significantly elevated concentrations of 5-oxoeicosapentaenoic acid (5-OxoEPA), cholesterol valerate, and docosahexaenoic acid-d5 (DHA-d5) in the *Brassica rapa* group (* *p* < 0.05).

Untargeted lipid metabolomic analysis of rumen fluid revealed marked differences between cows fed the *Brassica rapa L*. diet and those on the control diet. Volcano plot analysis identified a set of significantly upregulated lipid metabolites in the Bra group, indicating diet-induced shifts in lipid metabolism (Figure 2A). Network pathway mapping showed that many of these upregulated metabolites were positioned near liver X receptor alpha (LXRα) in the metabolic signaling landscape. Notably, metabolites such as cholesterol, oxidized fatty acids (e.g., 5-oxoeicosapentaenoic acid), diacylglycerols, and phospholipids were connected to LXRα and downstream regulators like SREBP-1c. This suggests that LXRα may act as a lipid-sensing node responsive to dietary fiber-derived microbial metabolites (Figure 2B). As shown in Figure 2C, Boxplot analysis confirmed that three LXR-related metabolites, 5-oxoeicosapentaenoic acid, cholesterol valerate, and docosahexaenoic acid-d5, were significantly elevated in the *Brassica rapa* group compared to the control, with all changes reaching statistical significance (*p* < 0.05). These data support a potential mechanistic link between dietary fiber intake, microbial lipid metabolism, and host transcriptional regulation through LXRα-mediated pathways.

### 3.3 Hypoxia-induced proteomic remodeling reveals LXR pathway activation

**Figure 3:**
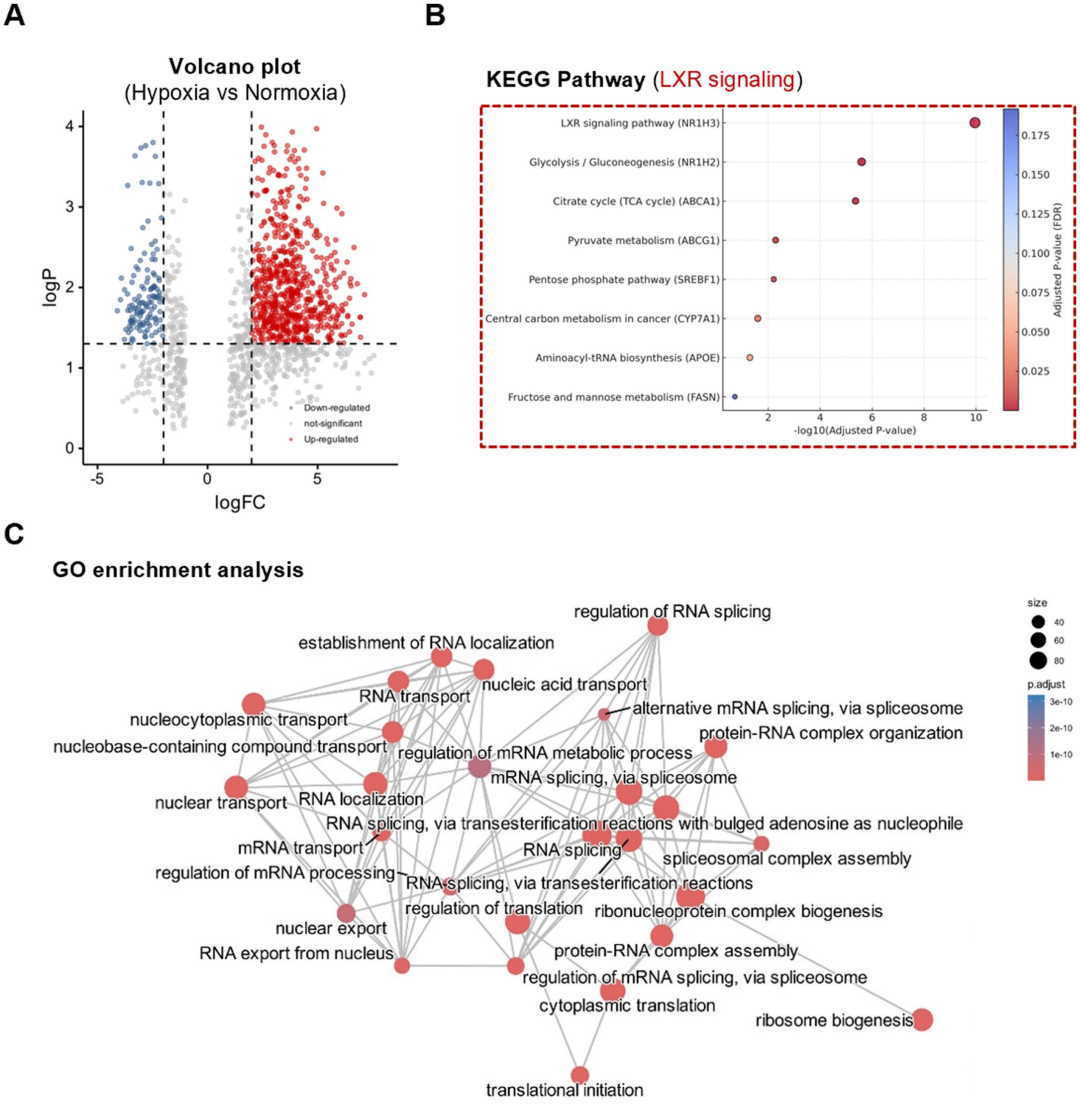
Hypoxia reprograms mammary epithelial cell proteome with enrichment of LXR-related lipid pathways. **(A)** Volcano plot showing significantly up- and downregulated proteins. **(B)** KEGG pathway enrichment of differentially expressed proteins under normoxia and hypoxia. **(C)** GO enrichment analysis highlights changes in lipid metabolism, circadian regulation, and VHL complex components. Significance threshold: fold change >1.5, *p* < 0.05.

To investigate how mammary epithelial cells adapt to hypoxia at the protein level, comparative proteomic analysis was performed between normoxic and hypoxic conditions. As shown in Figures 3A,B, hypoxia induced a distinct proteomic signature characterized by altered expression of multiple lipid metabolism-related proteins. These included key regulators of fatty acid biosynthesis, cholesterol homeostasis, and nuclear receptor activity. Functional enrichment analysis identified the LXR signaling pathway as one of the most significantly affected under hypoxia. Central metabolic regulators such as SREBP-1c, fatty acid synthase, and acetyl-CoA-related enzymes were upregulated and formed a tightly connected cluster around LXRα in the protein interaction network. Proteomic analysis revealed significant enrichment of oxidized lipid-binding proteins (e.g., FABP3, FABP5) and enzymes involved in cholesterol and triglyceride metabolism, including ACAT1, LIPA, and MGLL. These proteins are known to mediate intracellular lipid trafficking, esterification, and lipolysis, which are tightly regulated by nuclear receptors such as liver X receptor (LXRα). Their upregulation under hypoxic conditions suggests that mammary epithelial cells adapt to low oxygen by shifting toward lipid-storing and lipid-oxidizing pathways. This pattern aligns with the observed activation of LXRα signaling, which integrates oxidized lipid signals to coordinate gene expression in fatty acid biosynthesis and cholesterol efflux pathways. Thus, these proteomic signatures support a model in which hypoxia primes the mammary gland for enhanced sensitivity to lipid-derived metabolic signals. In the GO analysis (Figure 3C), significantly enriched biological processes included lipid metabolic process, fatty acid biosynthesis, and sterol regulatory region binding, indicating a strong functional orientation toward lipid regulation. These proteomic findings closely mirror the changes observed in the rumen metabolome of *Brassica rapa L*.-fed cows, where elevated levels of LXR-activating metabolites such as 5-oxo-eicosapentaenoic acid and cholesterol valerate were detected. The data suggest that mammary epithelial cells exhibit enhanced sensitivity to lipid-derived regulatory signals under hypoxic conditions, and that LXRα may function as a key metabolic integrator coordinating lactation-relevant responses to dietary and environmental factors.

### 3.4 Dietary metabolites and hypoxia synergistically induce LXRα expression and localization

**Figure 4:**
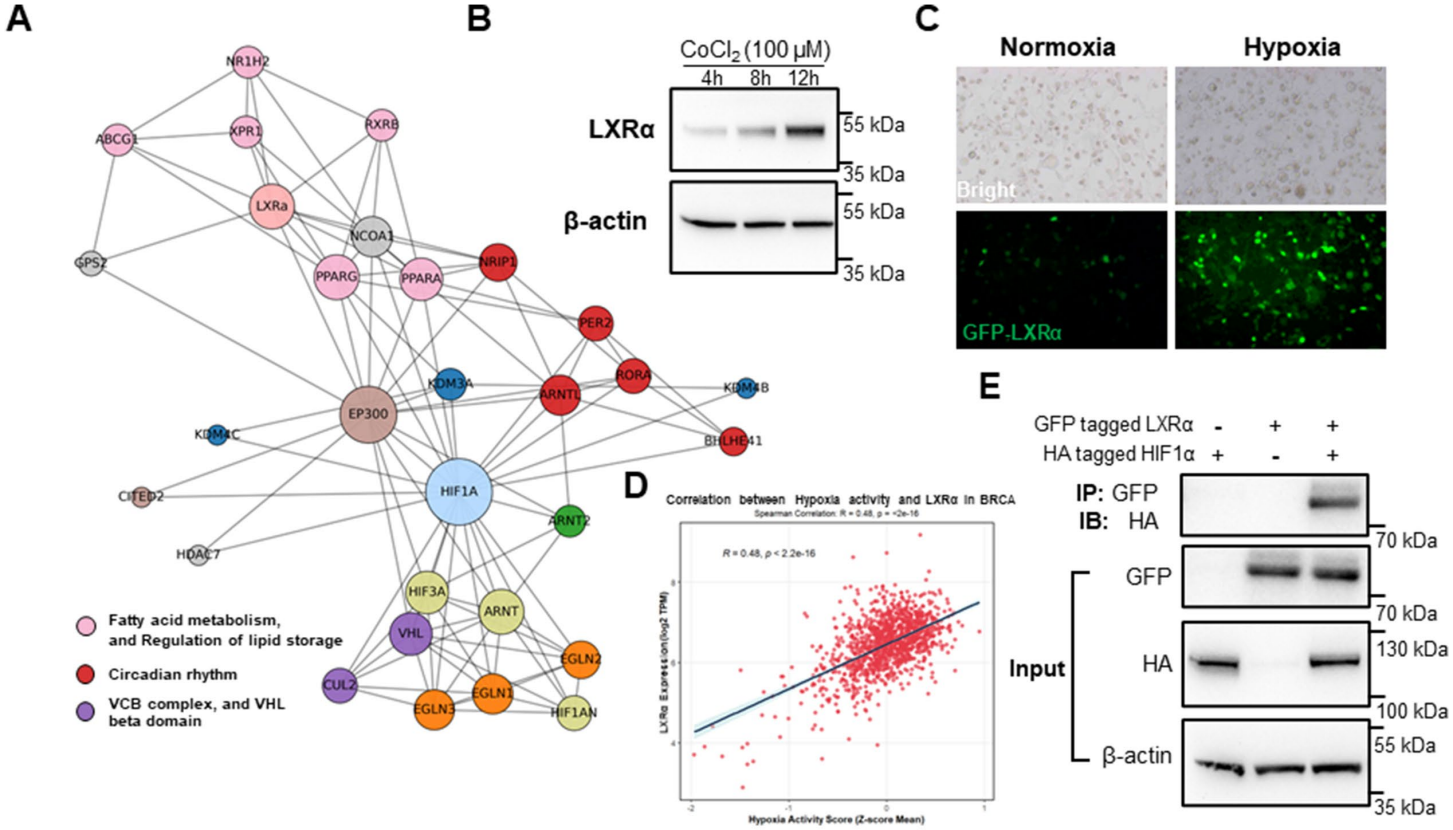
Dietary metabolites and hypoxia synergistically induce LXRα expression and localization. **(A)** Protein interaction network showing LXRα as a central node connecting fatty acid metabolism, circadian rhythm, and hypoxia response pathways. **(B)** Western blot analysis of LXRα expression in BME-UV1 cells under normoxia, hypoxia, at 4 h, 8 h, and 12 h following CoCl₂ exposure. **(C)** Immunofluorescence images of GFP-tagged LXRα showing nuclear localization under hypoxic conditions. **(D)** Correlation analysis of the TCGA-BRCA dataset showing a significant positive correlation between hypoxia activity score and NR1H3 (LXRα) expression. **(E)** Co-immunoprecipitation (Co-IP) reveals a direct interaction between LXRα and HIF-1α under hypoxic conditions.

As shown in Figure 4A, protein interaction network analysis revealed that LXRα (NR1H3) was closely associated with key transcriptional regulators of fatty acid metabolism (PPARG, SREBF1), circadian rhythm (PER2, RORA), and hypoxia response (HIF1A, EP300). The network also indicated connections to components of the VHL complex (e.g., VHL, CUL2), suggesting that lipid regulation under hypoxia is integrated with oxygen-sensing and ubiquitin-mediated degradation pathways. Hypoxia and CoCl₂ treatment significantly increased LXRα protein expression in BME-UV1 bovine mammary epithelial cells (Figure 4B). Time-course analysis confirmed a progressive upregulation of LXRα at 4 h and 8 h post-CoCl₂ exposure compared to normoxic conditions, indicating a hypoxia-induced response.

GFP-tagged LXRα showed marked nuclear accumulation under hypoxia (Figure 4C), consistent with activation of its transcriptional function. Under normoxia, GFP-LXRα was diffusely distributed in the cytoplasm, while hypoxia promoted clear nuclear translocation, demonstrating its activation under hypoxic conditions. Importantly, the correlation analysis based on human breast cancer transcriptomic data (BRCA, TCGA cohort) shows a significant positive association between hypoxia activity scores and NR1H3 (LXRα) gene expression (*R* = 0.48, *p* < 2.2 × 10^−16^, Figure 4D). This cross-species evidence supports the notion that LXRα expression is responsive to hypoxia in both bovine and human mammary cells, reinforcing its role as a convergence point for lipid signaling and environmental stress adaptation. To further investigate whether LXRα and HIF-1α interact at the protein level, we performed co-immunoprecipitation (Co-IP) assays in HEK293 cells transiently expressing tagged bovine LXRα and HIF-1α under hypoxia-mimicking conditions (CoCl₂, 100 μM, 8 h). As shown in Figure 4E, reciprocal pull-down confirmed a direct physical interaction between LXRα and HIF-1α. This result provides additional mechanistic evidence that LXRα activation under hypoxia may involve coordinated regulation or complex formation with HIF-1α.

### 3.5 A mechanistic model associates fiber intake with lactation via a microbiota-mammary axis

**Figure 5:**
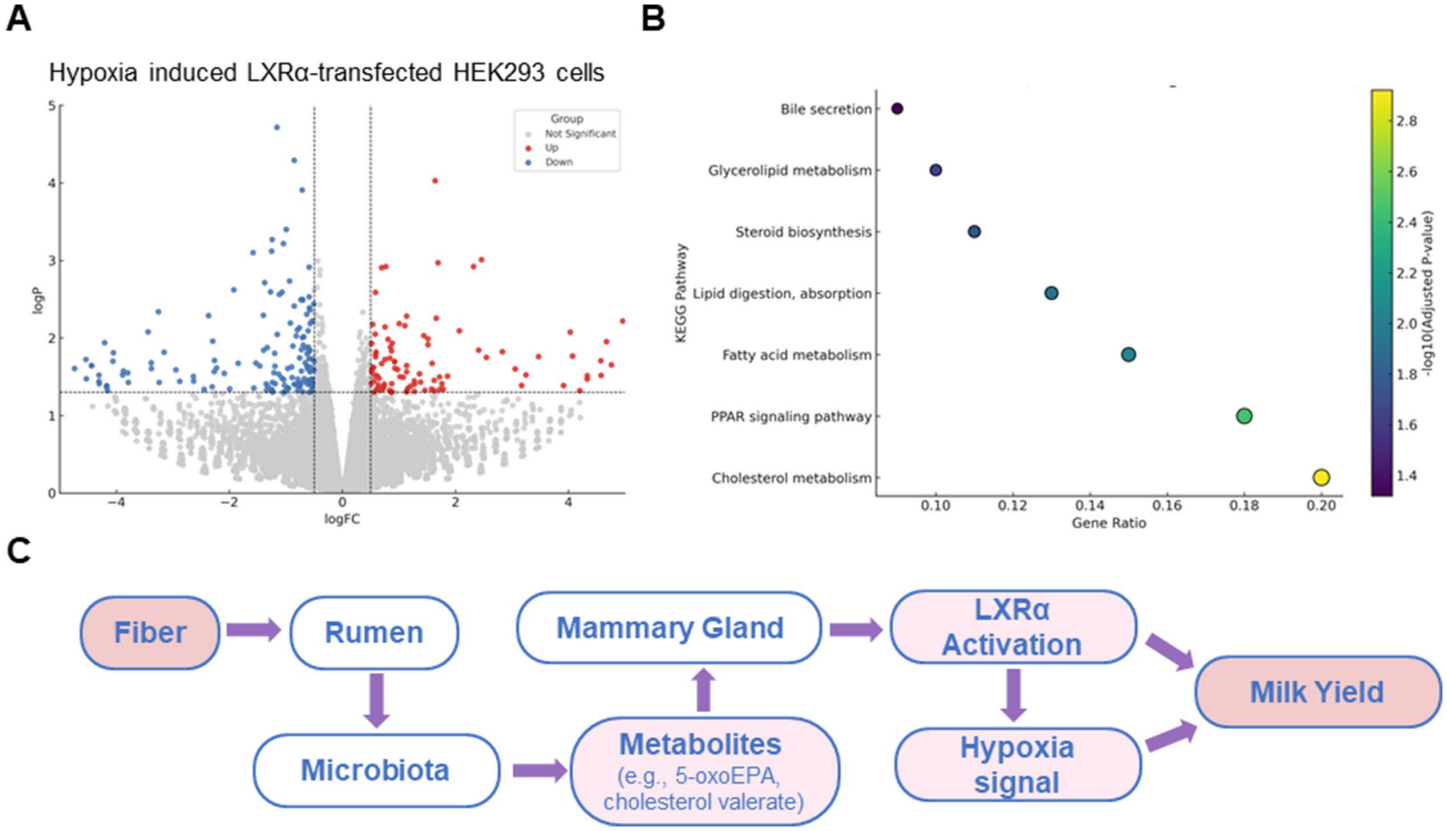
A mechanistic model associates fiber intake with lactation via a microbiota-mammary axis. **(A)** Volcano plot of differentially expressed genes in HEK293 cellss overexpressing bovine LXRα. **(B)** KEGG pathway enrichment of these genes shows involvement in cholesterol metabolism, fatty acid biosynthesis, PPAR signaling, and bile secretion. **(C)** Schematic model of the proposed “rumen microbiota–mammary gland axis.” This is a conceptual summary based on earlier metabolomic and proteomic findings in this study, rather than a presentation of new microbiota sequencing data.

To validate the transcriptional regulatory function of LXRα in a well-controlled system, we employed the HEK293 cell line for overexpression and transcriptomic analysis. HEK293 cells are widely used for transfection efficiency and transcriptome studies due to their stable growth and low endogenous expression of LXRα, making them suitable for exogenous gene function testing. This model allowed us to dissect the direct transcriptional targets and enriched metabolic pathways regulated by bovine LXRα, independent of confounding endogenous factors in mammary cells. As shown in Figure 5A, LXRα activation induced widespread transcriptional changes, with a large number of genes significantly upregulated (red) or downregulated (blue). These results indicate a strong regulatory influence of LXRα on global gene expression. KEGG pathway enrichment analysis of the differentially expressed genes revealed significant overrepresentation of pathways involved in lipid digestion and absorption, fatty acid metabolism, cholesterol metabolism, and PPAR signaling (Figure 5B). Notably, these pathways are functionally aligned with those enriched in the proteomic and metabolomic data obtained from rumen and mammary tissues, supporting a conserved role of LXRα in coordinating lipid-related processes. Based on these findings, a working model is proposed (Figure 5C) in which dietary fiber influences milk yield through a fiber-rumen-microbiota-mammary gland axis. In this model, fiber alters the rumen microbial community, leading to increased production of lipid-derived metabolites. These metabolites activate LXRα signaling in mammary epithelial cells, particularly under hypoxic conditions, enhancing lipid biosynthesis and supporting lactation.

## 4 Discussion

In this study, we demonstrated that supplementing plateau dairy cows with a high-fiber diet based on *Brassica rapa L.* significantly enhanced milk yield without increasing total feed intake. This indicates that the improvement in lactation performance was not due to increased nutrient consumption, but rather to the functional effects of fiber on rumen fermentation and systemic metabolism. Elevated levels of specific microbial-derived lipid metabolites in the rumen, including cholesterol valerate and 5-oxoeicosapentaenoic acid, suggest a metabolic linkage between fiber intake and mammary gland function under high-altitude conditions (14, 15).

The rumen is the primary site of microbial fermentation in ruminants, and its functional output determines energy availability for key physiological processes such as lactation (16, 17). Fiber intake promotes the proliferation of cellulolytic and saccharolytic bacteria that degrade complex plant polysaccharides into short-chain fatty acids and bioactive lipids (9, 18). Our metabolomic analysis shows that cows on the *Brassica rapa L.* diet produced significantly higher concentrations of bioactive lipids. These findings are consistent with previous studies reporting that fiber-enriched diets modulate microbial composition and metabolic output toward lipid metabolism pathways.

The systemic effects of these microbial metabolites are particularly relevant in hypoxia (19, 20). High-altitude environments such as the Qinghai-Tibet Plateau present chronic low-oxygen conditions that impair mammary gland metabolism. Hypoxia reduces oxygen delivery and limits ATP production, negatively affecting milk biosynthesis (21, 22). However, mammary epithelial cells retain adaptive capacity, as shown by our *in vitro* experiments under hypoxic conditions. Treatment with CoCl₂ induced upregulation and nuclear translocation of LXRα, a lipid-sensitive nuclear receptor that regulates lipogenic gene expression. Treatment with fiber-induced microbial metabolites further enhanced LXRα activation, supporting a synergistic role of dietary fiber and hypoxia in activating lactation-related signaling (23, 24). This supports the hypothesis that microbial metabolites derived from fiber fermentation act as endocrine-like molecules that help mammary cells adapt to hypoxic stress via LXR-dependent mechanisms.

The proteomic analysis confirmed that hypoxia remodeled the cellular metabolic landscape, upregulating proteins involved in lipid biosynthesis and nuclear receptor signaling. Enrichment of the LXR pathway, along with circadian rhythm and VHL complex-related proteins, indicates a complex regulatory network modulated by environmental and nutritional factors (25, 26). Activation of lipid-related pathways under hypoxia may represent an adaptive response of mammary tissue, which can be nutritionally supported through fiber-driven microbial signals. These findings provide a new perspective on how the diet-microbiome axis can be leveraged to improve milk production in environmentally challenging conditions. Beyond plateau dairy cows, the fiber–microbiota–LXRα regulatory axis may also be relevant to other ruminants such as yaks, goats, or sheep, especially those adapted to high-altitude or arid environments. These species share similar microbial fermentation systems and may benefit from nutritional strategies that modulate lipid-sensitive transcriptional pathways under environmental stress. Thus, fiber-targeted interventions could represent a broadly applicable approach to enhance lactation and metabolic resilience across diverse ruminant systems.

Nevertheless, this study has several limitations. Although we demonstrated a strong correlation between fiber intake, rumen metabolites, and LXR activation *in vitro*, *in vivo* evidence of this pathway in the mammary tissue remains to be confirmed. The transport dynamics of rumen-derived metabolites to the mammary gland were not directly measured, and receptor-ligand binding interactions remain to be biochemically validated (27, 28). In addition, while milk yield was increased, the effects on milk composition, such as fat and protein content, were not assessed. These aspects are critical for evaluating the commercial relevance of dietary interventions.

Future studies should focus on tracing labeled metabolites from the rumen to peripheral tissues to establish a direct metabolic link and quantify their endocrine-like effects. It is critical to investigate how different fiber types and sources affect microbial taxa and their metabolic products (29, 30). Integrated multi-omics approaches, including metabolomics, transcriptomics, and microbiome sequencing of mammary tissue, could provide a comprehensive view of the nutritional regulation of lactation. Moreover, field-scale trials are warranted to validate the application of fiber-based dietary strategies in high-altitude dairy production systems.

There are some limitations to this study that warrant mention. First, the mechanistic assays were performed in HEK293 cells rather than primary bovine cells, which, although selected for their transfection efficiency and low endogenous LXRα background, may not fully capture the complexity of native mammary cell responses. Second, the present study did not include long-term tracking of lactation performance. Future studies should consider *in vivo* validation of the microbiota–LXRα axis and explore whether breed-specific differences exist in the response to fiber-mediated interventions under hypoxia. These efforts would help confirm the physiological relevance and translational potential of the proposed mechanism.

## 5 Conclusion

In summary, supplementing plateau dairy cows with a fiber-rich diet significantly enhanced milk yield by improving rumen fermentation and reshaping microbial metabolic output. The *Brassica rapa L.* diet specifically increased the abundance of lipid-metabolizing microbiota, leading to the production of key metabolites such as cholesterol valerate and 5-oxoeicosapentaenoic acid. These compounds activated LXR signaling in hypoxic mammary epithelial cells, thereby promoting lipid biosynthesis and supporting lactation under environmental stress. In addition to enhancing fiber degradation in the rumen, this dietary intervention modulated systemic metabolism through the microbiota–mammary gland axis. Notably, we identified a direct interaction between LXRα and HIF-1α under hypoxic conditions, uncovering a previously unrecognized molecular link that integrates metabolic and oxygen-sensing pathways. These findings provide new mechanistic insights into lactation physiology and suggest that strategic fiber supplementation may support milk production and metabolic resilience in high-altitude ruminant systems.

## Data Availability

The data presented in the study are deposited in the Sequence Read Archive (SRA) under project number PRJNA1262543.

## References

[1] Lima J Martínez-Álvaro M Mattock J Auffret MD Duthie C-A Cleveland MA. Temporal stability of the rumen microbiome and its longitudinal associations with performance traits in beef cattle. Sci Rep. (2024) 14:20772. doi: 10.1038/s41598-024-70770-3 39237607 PMC11377694

[2] Guo W Zhou M Li F Neves ALA Ma T Bi S. Seasonal stability of the rumen microbiome contributes to the adaptation patterns to extreme environmental conditions in grazing yak and cattle. BMC Biol. (2024) 22:240. doi: 10.1186/s12915-024-02035-4 39443951 PMC11515522

[3] Kong Z Li B Zhou C He Q Zheng Y Tan Z. Multi-omics analysis of mammary metabolic changes in dairy cows exposed to hypoxia. Front Vet Sci. (2021) 8:764135. doi: 10.3389/fvets.2021.764135 34722715 PMC8553012

[4] Huang M Zhang X Yan W Liu J Wang H. Metabolomics reveals potential plateau adaptability by regulating inflammatory response and oxidative stress-related metabolism and energy metabolism pathways in yak. J Anim Sci Technol. (2022) 64:97–109. doi: 10.5187/jast.2021.e129 35174345 PMC8819316

[5] Chen H Wang C Huasai S Chen A. Effects of dietary forage to concentrate ratio on nutrient digestibility, ruminal fermentation and rumen bacterial composition in Angus cows. Sci Rep. (2021) 11:17023. doi: 10.1038/s41598-021-96580-5 34426627 PMC8382751

[6] Liu K Zhang Y Yu Z Xu Q Zheng N Zhao S. Ruminal microbiota–host interaction and its effect on nutrient metabolism. Anim Nutr. (2021) 7:49–55. doi: 10.1016/j.aninu.2020.12.001 33997331 PMC8110878

[7] Zhang Y Fan X Qiu L Zhu W Huang L Miao Y. Liver X receptor α promotes milk fat synthesis in buffalo mammary epithelial cells by regulating the expression of FASN. J Dairy Sci. (2021) 104:12980–93. doi: 10.3168/jds.2021-20596 34593221

[8] Wang B Tontonoz P. Liver X receptors in lipid signalling and membrane homeostasis. Nat Rev Endocrinol. (2018) 14:452–63. doi: 10.1038/s41574-018-0037-x 29904174 PMC6433546

[9] Makki K Deehan EC Walter J Bäckhed F. The impact of dietary Fiber on gut microbiota in host health and disease. Cell Host Microbe. (2018) 23:705–15. doi: 10.1016/j.chom.2018.05.012 29902436

[10] Wu J Yang D Gong H Qi Y Sun H Liu Y. Multiple omics analysis reveals that high fiber diets promote gluconeogenesis and inhibit glycolysis in muscle. BMC Genomics. (2020) 21:660. doi: 10.1186/s12864-020-07048-1 32972369 PMC7513505

[11] Yang X Wen D Liu Z Zhang Y Huang X Li B. Biofermentation of aquatic plants: potential novel feed ingredients for dairy cattle production. Sci Total Environ. (2024) 952:175955. doi: 10.1016/j.scitotenv.2024.175955 39222819

[12] Yang X Fan X Jiang H Zhang Q Zhang Q Dang S. Simulated seasonal diets alter yak rumen microbiota structure and metabolic function. Front Microbiol. (2022) 13:1006285. doi: 10.3389/fmicb.2022.1006285 36212853 PMC9538157

[13] Zhang Z Sun Y Zhong X Zhu J Yang S Gu Y. Dietary crude protein and protein solubility manipulation enhances intestinal nitrogen absorption and mitigates reactive nitrogen emissions through gut microbiota and metabolome reprogramming in sheep. Anim Nutr. (2024) 18:57–71. doi: 10.1016/j.aninu.2024.04.003 39035982 PMC11260031

[14] Yu Y Raka F Adeli K. The role of the gut microbiota in lipid and lipoprotein metabolism. J Clin Med. (2019) 8:2227. doi: 10.3390/jcm8122227 31861086 PMC6947520

[15] Serena C Ceperuelo-Mallafré V Keiran N Queipo-Ortuño MI Bernal R Gomez-Huelgas R. Elevated circulating levels of succinate in human obesity are linked to specific gut microbiota. ISME J. (2018) 12:1642–57. doi: 10.1038/s41396-018-0068-2 29434314 PMC6018807

[16] Cammack KM Austin KJ Lamberson WR Conant GC Cunningham HC. RUMINANT NUTRITION SYMPOSIUM: tiny but mighty: the role of the rumen microbes in livestock production. J Anim Sci. (2018) 96:752–70. doi: 10.1093/jas/skx053 29385535 PMC6140983

[17] Newbold CJ Ramos-Morales E. Review: ruminal microbiome and microbial metabolome: effects of diet and ruminant host. Animal. (2020) 14:s78–86. doi: 10.1017/S1751731119003252 32024572

[18] Fu J Zheng Y Gao Y Xu W. Dietary Fiber intake and gut microbiota in human health. Microorganisms. (2022) 10:22507. doi: 10.3390/microorganisms10122507 PMC9787832 36557760

[19] Wu J Wang K Wang X Pang Y Jiang C. The role of the gut microbiome and its metabolites in metabolic diseases. Protein Cell. (2021) 12:360–73. doi: 10.1007/s13238-020-00814-7 33346905 PMC8106557

[20] Spivak I Fluhr L Elinav E. Local and systemic effects of microbiome-derived metabolites. EMBO Rep. (2022) 23:e55664. doi: 10.15252/embr.202255664 36031866 PMC9535759

[21] Jin Y Liu Z Yang Z Fang L Zhao F-Q Liu H. Effects of hypoxia stress on the milk synthesis in bovine mammary epithelial cells. J Anim Sci Biotechnol. (2025) 16:37. doi: 10.1186/s40104-025-01174-0 40050971 PMC11887346

[22] Reddan B Cummins EP. The regulation of cell metabolism by hypoxia and hypercapnia. J Biol Chem. (2025) 301:108252. doi: 10.1016/j.jbc.2025.108252 39914740 PMC11923829

[23] Zhao L Zhang F Ding X Wu G Lam YY Wang X. Gut bacteria selectively promoted by dietary fibers alleviate type 2 diabetes. Science. (2018) 359:1151–6. doi: 10.1126/science.aao5774 29590046

[24] Fagundes RR Belt SC Bakker BM Dijkstra G Harmsen HJM Faber KN. Beyond butyrate: microbial fiber metabolism supporting colonic epithelial homeostasis. Trends Microbiol. (2024) 32:178–89. doi: 10.1016/j.tim.2023.07.014 37596118

[25] Duong HA Baba K DeBruyne JP Davidson AJ Ehlen C Powell M. Environmental circadian disruption re-writes liver circadian proteomes. Nat Commun. (2024) 15:5537. doi: 10.1038/s41467-024-49852-3 38956413 PMC11220080

[26] Russo-Savage L Schulman IG. Liver X receptors and liver physiology. Biochim Biophys Acta Mol basis Dis. (2021) 1867:166121. doi: 10.1016/j.bbadis.2021.166121 33713792 PMC9242550

[27] Wang YJ Xiao JX Li S Liu JJ Alugongo GM Cao ZJ. Protein metabolism and signal pathway regulation in rumen and mammary gland. Curr Protein Pept Sci. (2017) 18:636–51. doi: 10.2174/1389203717666160627075021 27356938

[28] Huang Q Xiao Y Sun P. Rumen–mammary gland axis and bacterial extracellular vesicles: exploring a new perspective on heat stress in dairy cows. Anim Nutr. (2024) 19:70–5. doi: 10.1016/j.aninu.2024.08.003 39628643 PMC11612815

[29] Khorasaniha R Olof H Voisin A Armstrong K Wine E Vasanthan T. Diversity of fibers in common foods: key to advancing dietary research. Food Hydrocoll. (2023) 139:108495. doi: 10.1016/j.foodhyd.2023.108495

[30] Ye S Shah BR Li J Liang H Zhan F Geng F. A critical review on interplay between dietary fibers and gut microbiota. Trends Food Sci Technol. (2022) 124:237–49. doi: 10.1016/j.tifs.2022.04.010

